# Human Metapneumovirus in Children, Singapore

**DOI:** 10.3201/eid1309.070220

**Published:** 2007-09

**Authors:** Liat Hui Loo, Boon Huan Tan, Ley Moy Ng, Nancy W.S. Tee, Raymond T.P. Lin, Richard J. Sugrue

**Affiliations:** * Nanyang Technological University, Singapore; † Kandang Kerbau Women’s and Children’s Hospital, Singapore; ‡ DSO National Laboratories, Singapore; § National University Hospital, Singapore

**Keywords:** Human metapneumovirus, paramyxovirus, P protein, dispatch

## Abstract

Four hundred specimens were collected from pediatric patients hospitalized in Singapore; 21 of these specimens tested positive for human metapneumovirus (HMPV), with the A2 genotype predominating. A 5% infection rate was estimated, suggesting that HMPV is a significant cause of morbidity among the pediatric population of Singapore.

*Human metapneumovirus* (HMPV) is a new member of the family *Paramyxoviridae*. It was first identified in children with respiratory diseases in the Netherlands and is now recognized as a substantial cause of acute respiratory infection in pediatric patients (*1*). The clinical symptoms in children are similar to those observed during respiratory syncytial virus (RSV) infections and vary from upper respiratory tract infection (URTI) to bronchiolitis and pneumonia. HMPV infections have been detected in young children 5 years of age (*2*) as well as in adults of all age groups (*3*). Sequence analysis of HMPV isolates has identified 2 main lineages, A and B; each group is further subdivided into 2 more lineages, A1 and A2, and B1 and B2 (*4**,**5*). Both virus genotypes were reported in various countries in the Americas, Europe, and Asia. This study aims to assess the importance of HMPV infection among hospitalized pediatric patients in Singapore.

## The Study

Kandang Kerbau Women’s and Children’s hospital is one of the major centers in Singapore for the admission of sick children, including those showing respiratory illness. After obtaining prior approval from the hospital’s ethics committee (approval number EC/043/2004), we collected nasopharyngeal swabs from 400 pediatric patients between October 2005 and January 2007. When admitted to the hospital, these patients exhibited symptoms of acute lower respiratory tract infections (LRTI) (bronchiolitis, bronchitis, pneumonia, asthma, and wheezing) and URTI (pharnygitis). Specimens were sent to the hospital’s microbiologic laboratory for routine testing for influenza A and B viruses, RSV, adenovirus, and parainfluenza virus (serotypes 1–3) by immunofluorescence assay (LIGHTDIAGNOSTICS, Chemicon, Tamacula, CA, USA). The clinical specimens were stored at –80^o^C until further analysis for HMPV was performed (not longer than a week after collection). Viral RNA (vRNA) was extracted from each of the thawed nasopharyngeal swabs with the QIAamp viral RNA minikit (QIAGEN Inc., Valencia, CA, USA) according to the manufacturer’s instructions. Of the total RNA extracted from the clinical specimens, 5 μL was subjected to real-time reverse transcription–PCR (RT-PCR) testing by using the N gene specific primer set NL–N (*6*). This was performed with the OneStep RT–PCR kit (QIAGEN) on a Corbett Research Rotorgene 3000 (Corbett Life Science, Sydney, NSW, Australia). The PCR cycling conditions were 50^o^C for 30 min, 95^o^C for 15 min, and 45 cycles (95^o^C for 20 s and 60^o^C for 60 s). Specimens that tested positive by real-time RT-PCR analysis were confirmed by conventional RT–PCR by using the NL–N primer set. The amplified products (163 bp) were detected by using agarose gel electrophoresis, and their identity was confirmed by DNA sequencing.

Of the 400 samples collected, 21 tested positive for HMPV infection, which suggests an incidence rate of ≈5.3%, compared with an 11.5% incidence rate for RSV (Table 1). Previous reports have suggested that in some cases severe symptoms exhibited by RSV-infected patients are associated with dual infections involving HMPV (*7*). Although we detected the presence of HMPV and RSV in the patients screened, no evidence for co-infections was observed, which suggests a low occurrence for these viruses in Singapore. In a recent study in Australia, only 8 of 10,000 screened hospitalized patients showed evidence of co-infection with HMPV and RSV (*8*). In contrast, several recent studies suggest that co-infections may account for a substantial number of instances in which HMPV has been detected. For example, a recent study in Brazil, which used a lower sample size than in our study, reported an 8% incidence rate for pediatric patients who had RSV and HMPV co-infections (*9*). Therefore, environmental factors may be a key feature in the development of co-infections.

**Table 1:** Positive test results for respiratory viruses from clinical specimens (n = 400)

Virus	No. positive (%)
Respiratory syncytial virus	46 (11.5)
Influenza A virus	3 (0.8)
Influenza B virus	1 (0.3)
Parainfluenza 1 virus	4 (1.0)
Parainfluenza 2 virus	0 (0)
Parainfluenza 3 virus	8 (2.0)
Adenovirus	1 (0.3)
Human metapneumovirus	21 (5.3)
Total	84 (21.0)

The entire P gene sequences were amplified directly from the specimens by RT–PCR using the primers hmptPF 5′-ATGTCGTTCCCTGAAGGAAAAGATATTC-3′ and hmptPR 5′-TTAAACTACATAATTAAGTGGTAAAT-3′. Amplicons 884 bp in size were generated and corresponded to 1209 nt–2093 nt of the HMPV genome (strain JPS03-240, AY530095). PCR cycling was performed on a conventional thermal cycler by using a “touch-down” procedure; conditions were 94^o^C for 5 min followed by 30 cycles of 94^o^C for 15 s, 62^o^C (reducing by 0.5^o^C/cycle) for 30 s, 72^o^C for 1 min, and a final extension step of 72^o^C for 7 min. The sizes of the respective PCR-amplified products were examined by using agarose gel electrophoresis, gel-purified, and confirmed by DNA sequencing. The sequences were submitted to GenBank under accession nos. EF409351–EF409371. The genetic relationship between the Singapore HMPV isolates and those HMPV isolates described previously was analyzed by comparing the P gene sequences (*10*). Alignments of nucleic acid sequences were created by using ClustalX version 1.83 (bips.u-strasbg.fr/fr/documentation/clustalx). Phylogenetic trees were constructed by using the neighbor-joining method (1,000 bootstrap replicates) and edited with MEGA 3.1 (*11*). Comparisons were made with representatives of the 4 genetic lineages (Figure). This analysis shows that although isolates representing both A and B genotypes were detected, the Singapore isolates clustered more predominantly with representative HMPV strains in lineage A, in particular the sublineage A2. In this study HMPV was detected throughout the year, which suggests that in Singapore, HPMV is present in the pediatric community throughout the year. We also noted a slight increase in the incidence of B genotypes (B1 and B2) during the last quarter of 2006, but the implications of this finding are unclear.

**Figure:**
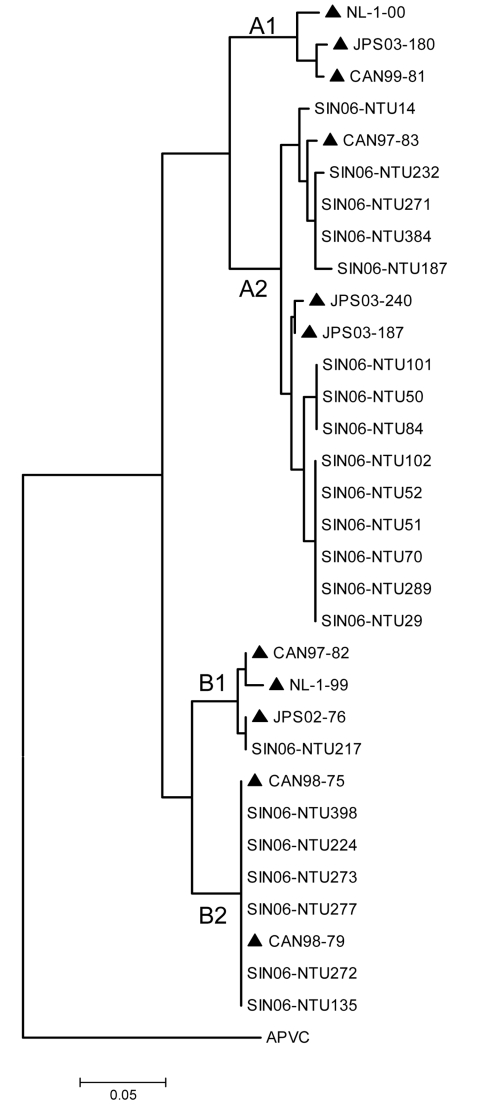
Phylogenetic analyses of nucleotide sequences of HMPV phosphoprotein showing comparisons with Singapore-Nanyang Technological University (SIN06-NTU*) sequences. *The specimen number acquired during the course of the investigation (e.g., SIN06-NTU14) was made with known strains (highlighted ▲) from Canada [CAN99-81 (AY145294, AY145249), CAN97-83 (AY297749), CAN97-82 (AY145295, AY145250), CAN98-75 (AY297748), CAN98-79 (AY145293, AY145248)], Japan [JPS03-180 (AY530092), JPS03-240 (AY530095), JPS03-187 (AY530093), JPS02-76 (AY530089)], and the Netherlands [NL-1-00 (AF371337), NL-17-00 (AY304360), NL-1-99 (AY525843), NL-1-94 (AY304362)]. Avian pneumovirus type C (APVC AY590688) was used as the outgroup.

The age and clinical characteristics of the HMPV patients were next compared with the different HMPV lineages (Table 2). Children with HMPV infection were 1 month to 12 years in age; 67% were <1 year of age compared with 63% of RSV-infected children. Of the HMPV-infected patients, 52% exhibited LRTI; of these, 82% were infected with the HMPV sublineage A2. In contrast, ≈43% of the patients exhibited URTI caused by the sublineages A2 and B2. In comparison, 61% and 20% of the RSV patients had a clinical diagnosis of LRTI or URTI, respectively. Our data suggested an increased association of sublineage A2 with LRTI in the HMPV-infected patients. The implications of this are unclear, but several reports note a correlation between severity of infection and the presence of the A genotype (*12**,**13*). Unfortunately, we were not able to make a strict comparison of our data with data from recent studies in Southeast Asia (*14**,**15*); these studies used significantly smaller sample sizes and a different selection criterion for the patients screened (i.e., LRTI [*14*] and wheezing and asthma [*15*]).

**Table 2:** Characteristics of pediatric patients with human metapneumovirus (HMPV) infection

Patient no.	Age	Clinical diagnosis*	Lineage of HMPV
14	2 y	L	A2
29	1 y	O	A2
50	6 mo	U	A2
51	1 y	L	A2
52	1 y	U	A2
70	11 mo	U	A2
84	6 mo	U	A2
101	1 y	L	A2
102	2 y	L	A2
135	1 y	U	B2
187	3 mo	L	A2
217	1 mo	L	B1
224	1 y	U	B2
232	1 y	L	A2
271	12 y	L	A2
272	4 y	U	B2
273	1 y	U	B2
277	5 y	U	B2
289	1 y	L	A2
384	2 y	L	A2
398	7 y	L	B2

*L, lower respiratory infections including bronchiolitis, bronchitis, pneumonia, asthma, wheezing or chest infection; U, upper respiratory infections including infantile pyrexia and pharyngitis; O, febrile fit.

## Conclusions

Our study is the first, to our knowledge, that has attempted to assess the importance of HMPV among the pediatric population in Singapore. We analyzed 400 samples that were collected from pediatric patients who were admitted to a hospital over a 16-month period. An infection rate of 5.3% was observed, which is consistent with the reported infection rates of several other industrialized countries. We also noted that of the viruses detected, ≈67% were of the A subtype and 33% were of the B subtype, which suggests that the former was the predominant HMPV subtype causing illness in these patients. Furthermore, a significant proportion of the HMPV-infected patients had LRTI. Our findings suggest that HMPV is a substantial cause of illness among the pediatric population of Singapore.
